# Eating Habits and Possible Effects of Mediterranean Diet on Body Composition in Ageing People Living with HIV in Two Italian Centres

**DOI:** 10.3390/v18090931

**Published:** 2026-08-25

**Authors:** Maria Mazzitelli, Giacomo Berti, Valentina Camozzi, Elisa Mazza, Samuele Gardin, Chiara Davoli, Chiara Costa, Vincenzo Scaglione, Tiziana Montalcini, Enrico Maria Trecarichi, Annamaria Cattelan, Carlo Torti

**Affiliations:** 1 Dipartimento di Sicurezza e Bioetica—Sezione di Malattie Infettive, Università Cattolica del Sacro Cuore, 00168 Rome, Italy; 2 Dipartimento di Scienze Mediche e Chirurgiche, Fondazione Policlinico Universitario Agostino Gemelli IRCCS, 00168 Rome, Italy; 3 Infectious and Tropical Diseases Unit, Padua University Hospital, 35128 Padua, Italy; 4 Unit of Clinical Research, Padua University Hospital, 35128 Padua, Italy; 5 Department of Medicine DIMED, Endocrine Unit, University of Padova, 35128 Padua, Italy; 6 Research Center for the Prevention and Treatment of Metabolic Diseases, University “Magna Græcia” of Catanzaro, 88100 Catanzaro, Italy; 7 Infectious and Tropical Diseases Unit, Azienda Ospedaliero-Universitaria “Renato Dulbecco”, 88100 Catanzaro, Italy; 8 Department of Clinical and Experimental Medicine, University “Magna Græcia” of Catanzaro, 88100 Catanzaro, Italy; 9 Dipartimento di Scienze Della Vita, Della Salute e Delle Professioni Sanitarie, Link Campus University, 00165 Rome, Italy

**Keywords:** people with HIV, Mediterranean diet, body composition, DXA, ageing, comorbidities, fat distribution

## Abstract

Background: The relationship between diet and body composition in people with HIV (PWH) is still not well defined. Therefore, our primary objective was to assess the association between Mediterranean diet (MD) adherence and body composition measured by whole-body dual-energy X-ray absorptiometry (DXA). Secondly, we aimed to assess differences between people living in the north (Padua, Veneto Region, Italy) vs. those living in the south of Italy (Catanzaro, Calabria Region, Italy) and with respect to ethnicity. Methods: In this cross-sectional study, adults with HIV with a confirmed virological suppression were enrolled at two Italian HIV clinics. MD adherence was assessed using the validated MedDiet Score, with high adherence being defined as a score ≥ 25. All participants underwent whole-body DXA. Anthropometric and DXA-derived measures were compared between people with high vs. low adherence to MD, and multivariable linear regression adjusted for age, sex, and CD4^+^ count at nadir was performed. Results: Among 130 participants (81.5% male; median age 57 years), 105 (80.8%) showed high MD adherence. High adherence to MD was more common among participants from Southern Italy than among those from Northern Italy and was associated with Caucasian ethnicity, higher education, higher CD4^+^ count and CD4/CD8 ratio, and a lower prevalence of obesity and neurological disorders. DXA analyses showed lower total and trunk fat mass and higher lumbar spine bone mineral density in highly adherent individuals. After adjustment, high MD adherence remained independently associated with lower BMI (β −5.3, 95% CI −7.3 to −3.4; *p* < 0.001). Conclusions: In ageing PWH, high MD adherence was associated with a more favourable clinical profile and healthier body composition, particularly lower central adiposity and BMI. Prospective and interventional studies are needed to clarify the role of this type of diet in PWH.

## 1. Introduction

With the widespread availability of effective antiretroviral therapy (ART), people with HIV (PWH) are living longer and increasingly experiencing age-related comorbidities, including non-AIDS-related cancers, obesity, dyslipidaemia, type 2 diabetes, metabolic dysfunction-associated steatosis liver disease, cardiovascular disease, osteoporosis, sarcopenia, and frailty, often requiring a multidimensional approach [1,2,3,4]. This evolving clinical phenotype reflects the combined impact of traditional risk factors (e.g., smoking, sedentary behaviour, suboptimal diet), persistent immune activation and low-grade inflammation despite viral suppression, and both short- and long-term ART-associated metabolic effects [5,6,7]. In particular, changes in adipose tissue distribution and muscle mass—together with reduced bone mineral density (BMD)—may cluster to amplify cardiometabolic and functional risk while also affecting quality of life and long-term prognosis [8].

Over the last few years, a great interest has emerged in lifestyle modifications in PWH, with increasing attention paid to modifiable factors (diet, exercise, smoking sleep quality) associated with increased cardiovascular risk [9,10,11]. In particular, diet quality represents a potentially modifiable determinant of cardiometabolic risk and inflammation in PWH. The Mediterranean diet (MD), characterised by a high consumption of vegetables, fruit, legumes, whole grains, nuts, and extra-virgin olive oil; moderate intake of fish and dairy; low intake of red/processed meat and sweets; and (when culturally appropriate) moderate wine consumption, has been consistently linked to improved metabolic profiles and reduced cardiovascular events in the general population, including in randomised trials [12,13]. MD patterns may influence insulin sensitivity, lipid metabolism, gut microbiota composition, and inflammatory pathways—domains that are particularly relevant in the context of HIV, where residual inflammation and metabolic dysregulation remain common [14,15]. In PWH, dietary patterns may also have broader implications for long-term health. Healthy dietary patterns, such as MD, have been associated with anti-inflammatory effects and improved immune function in the general population, suggesting that they may also support healthier ageing and reduce cardiometabolic risk in PWH. Although these potential mechanisms were not directly investigated, they provide an additional rationale for evaluating adherence to healthy dietary patterns in this population. Also, in PWH, observational evidence suggests that higher MD adherence is associated with more favourable anthropometric and metabolic parameters and may mitigate ART-related metabolic complications and inflammation [16]. However, reliance on body mass index alone can be misleading in HIV, because cardiometabolic risk is strongly influenced by fat distribution (particularly central/visceral adiposity) and by the concomitant loss of lean mass [17]. In this context, whole-body dual-energy X-ray absorptiometry (DXA) provides a robust, relatively low-radiation method to quantify total and regional body composition, including total fat mass, android and gynoid fat distribution, trunk and limb fat, and appendicular lean mass as a proxy for sarcopenia-related phenotypes [18]. Importantly, DXA simultaneously yields BMD estimates, allowing for an integrated assessment of adiposity, lean tissue, and skeletal health within a single examination and enabling an exploration of trade-offs (e.g., higher body fat with preserved BMD versus sarcopenic adiposity with low BMD) that may be clinically meaningful in ageing people with HIV [19,20].

Despite these advantages, data linking MD adherence to objective DXA-derived body composition measures—particularly central adiposity, lean mass, and BMD—remain limited, and potential differences related to geographic context within a Mediterranean country such as Italy have not been adequately explored. Therefore, the primary objective of our study was to investigate in a cross-sectional evaluation the association between adherence to the Mediterranean diet and body composition assessed by DXA in ageing PWH. The comparison between participants from Northern and Southern Italy was conceived as a secondary, exploratory objective, aimed at evaluating whether geographical context influenced dietary habits and whether it affected the observed associations.

## 2. Materials and Methods

### 2.1. Study Design and Setting

We performed a cross-sectional study in two Italian HIV clinics (Azienda Ospedale–Università di Padova, Northern Italy; Azienda Ospedaliero-Universitaria “Renato Dulbecco”, Catanzaro, Southern Italy). This study was conducted in accordance with the Declaration of Helsinki and the Principles of Good Clinical Practice. The protocol was approved by the relevant Ethics Committees of each participating centres (protocol IDs: MED-HIV 03/2018 and BEHAVE/2021-AOP1763), and all participants provided written informed consent for their participation in this study.

### 2.2. Inclusion Criteria and Study Procedures

Adult (age > 18 years) PWH attending routine outpatient follow-ups at both clinics were eligible if they had stable virological suppression (two HIV-RNA measurements in 6 months < 50 copies/mL). Exclusion criteria were the absence of informed consent, pregnancy, detectable HIV-RNA, HIV diagnosis < 6 months (to avoid the possible interference of body composition in the “return to health” phenomenon) and any condition preventing whole-body dual-energy X-ray absorptiometry (DXA) scan acquisition. Dietary habits were assessed using a validated food frequency questionnaire (MedDiet Score; see Table S1) capturing Mediterranean food consumption and dietary habits [21]. High adherence to MD was defined by a questionnaire score ≥ 25. On the same day as the questionnaire, all participants underwent a whole-body DXA scan. Whole-body DXA examinations were performed at each participating centre using the Hologic^®^ DXA system (Hologic, Inc., Marlborough, MA, USA) with Advanced Body Composition^®^ (2017 software). The scanners underwent routine daily calibration using the manufacturer’s calibration phantom according to standard operating procedures. Quality control procedures were performed regularly in accordance with the manufacturer’s recommendations. All scans were acquired and analysed by trained operators following standardised protocols. We analysed anthropometrics (weight, height, BMI, waist circumference) and DXA-derived measures, including total fat mass (kg), trunk fat mass (kg and %), android and gynoid fat measures, lean mass, and bone mineral density (BMD) (total body, femur, and lumbar spine). All participants also underwent liver transient elastography to assess the prevalence and grading of fatty liver disease and a blood test to assess viro-immunological and metabolic parameters.

### 2.3. Data Collection and Statistical Analysis

We recorded demographic characteristics (age, sex, ethnicity, educational level), lifestyle factors (smoking, alcohol use, physical activity), HIV-related variables (risk factor for acquisition, years living with HIV, prior AIDS event, CD4^+^ nadir, current CD4^+^ count, CD4/CD8 ratio, ART regimen), and comorbidities (including chronic obstructive pulmonary disease, COPD, dyslipidaemia, diabetes, hypertension, obesity (i.e., BMI > or = 30, as per WHO definition), and osteopenia/osteoporosis/fracture history). There were no missing data for any of the variables analysed. Continuous variables were summarised as the median (IQR) and compared using the Wilcoxon rank-sum test; categorical variables were compared using Pearson’s χ^2^ or Fisher’s exact test, as appropriate; ordinal categorical variables were compared using the Wilcoxon rank-sum test, treating ordered categories as ranks. We fitted multivariable linear regression models to assess the independent association of MD adherence and geographic origin with BMI, BMD, and body fat percentage, adjusting for age, sex, and CD4^+^ count at nadir. Two-sided *p*-values < 0.05 were considered statistically significant. All statistical analyses were performed using R version 4.5.0.

## 3. Results

Between December 2018 and December 2024, 130 PWH were enrolled, including 78 (60.0%) from Northern Italy and 52 (40.0%) from Southern Italy. Median age was 57.4 years (IQR: 48.3–69.3), and 106 participants (81.5%) were male. The median CD4^+^ count at the evaluation was 784.5 cells/µL (IQR: 617–1039), and the median CD4/CD8 ratio was 0.96 (IQR: 0.7–1.3).

Overall, 105 (80.8%) and 25 (19.2%) participants had high and low adherence to MD, respectively. The low-adherence group was predominantly composed of participants from Northern Italy (22/25, 88.0%), and this proportion was significantly lower in the high-adherence group (56/105, 53.3%; *p* = 0.001). By region, 49/52 (94.2%) participants from Southern Italy had high MD adherence, compared with 56/78 (71.8%) from Northern Italy. Ethnicity differed markedly by adherence: all participants with high adherence were Caucasian, whereas 10/25 (40.0%) in the low-adherence group were of non-Caucasian ethnicity (*p* < 0.001). High adherence was also associated with higher educational level (40.0% vs. 16.0%; *p* = 0.024) and a higher prevalence of current smoking (52.4% vs. 28.0%; *p* = 0.047). HIV-related immune status differed by adherence: participants with high adherence had higher current CD4^+^ counts (median 832 vs. 636 cells/µL; *p* = 0.021) and higher CD4/CD8 ratios (median 1.05 vs. 0.75; *p* = 0.018). Obesity was substantially less prevalent in the high-adherence group (19/105, 18.1%) than in the low-adherence group (18/25, 72.0%; *p* < 0.001). Neurological disorders were also less frequent with high adherence (1.0% vs. 12.0%; *p* = 0.022). Other comorbidities and ART regimen distributions were broadly comparable between groups (Table 1).

Overall, 39/130 (30%) people presented an alcohol use disorder in their history. Looking at the details of alcohol consumption (Table S2), about one out of three participants (32%) disclose of being a teetotal, and 46 (35.4%) people were classified by the survey as having a high-risk alcohol consumption. The high-risk alcohol consumption was significantly higher in people with high adherence to MD, and in people of Caucasian ethnicity.

**Figure 1:**
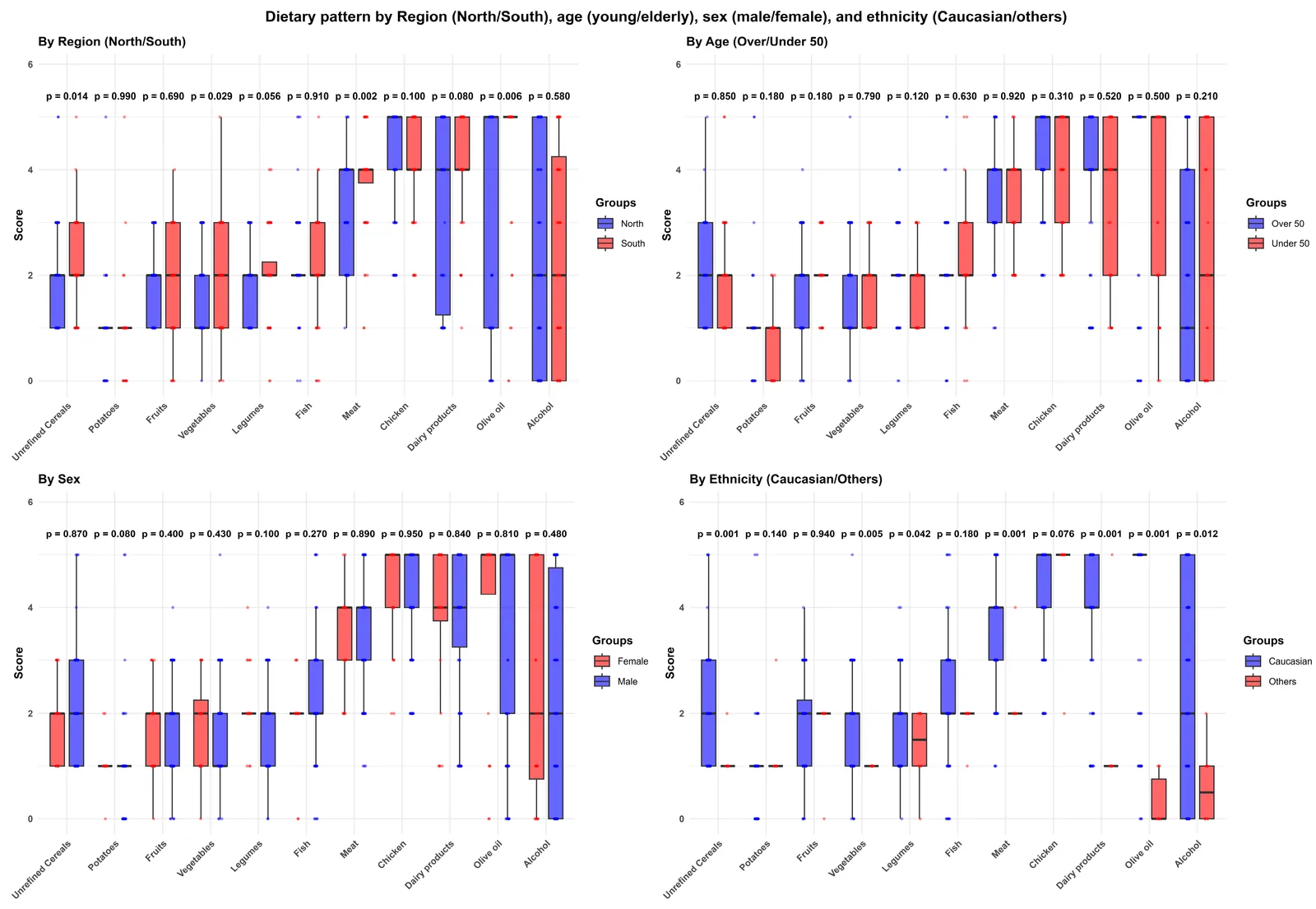
Dietary pattern by region, age, sex, and ethnicity. For each panel, boxes represent the interquartile range (IQR) of the dietary score for the two comparison groups (blue and red, as shown in the legend) with the horizontal line indicating the median; whiskers extend to the most extreme values within 1.5 × IQR, and individual data points are overlaid to show the full distribution, including outliers. The y-axis reports the dietary score, and the x-axis lists the dietary components (alcohol, meat, unrefined cereals, fruits, legumes, olive oil, potatoes, fish, chicken, dairy products, vegetables). *p*-values above each food group indicate the between-group comparison for that specific item. A scoring key is provided at the bottom: 0 = never; 1 = 1–6 times/week; 2 = 7–12; 3 = 13–18; 4 = 19–31; 5 = ≥32 times/week.

In Figure 1, regional comparisons (north vs. south) showed statistically significant differences in consumption scores for meat (*p* = 0.002), unrefined cereals (*p* = 0.014), olive oil (*p* = 0.006), and vegetables (*p* = 0.029), with no significant differences for the remaining components. Comparisons by age (≥50 vs. <50 years) and by sex (female vs. male) did not show significant differences across food components (all *p* > 0.05). In contrast, ethnicity (Caucasian vs. other) was associated with significant differences in multiple components, including alcohol (*p* = 0.012), meat (*p* = 0.001), unrefined cereals (*p* = 0.001), legumes (*p* = 0.042), olive oil (*p* = 0.001), dairy products (*p* = 0.001), and vegetables (*p* = 0.005), with a trend for chicken (*p* = 0.076) consumption.

Participants with high MD adherence had lower body weight (median 74.4 vs. 88.0 kg; *p* < 0.001) and lower BMI (median 25.59 vs. 31.38 kg/m^2^; *p* < 0.001) than those with low adherence, while waist circumference did not differ significantly (median 96.5 vs. 98.0 cm; *p* = 0.51).

At the DXA scan, high adherence was associated with lower adiposity, particularly central fat. Total fat mass was lower in the high-adherence group (median 18.606 vs. 21.921 kg; *p* = 0.009), as were trunk fat mass (median 11.471 vs. 15.934 kg; *p* = 0.002) and trunk fat percentage (median 31.1% vs. 35%; *p* = 0.004). Android fat measures were also lower in the high-adherence group in terms of both grams (median 1.930 vs. 3.107 kg; *p* = 0.001) and percentage (median 33.2% vs. 39.8%; *p* = 0.001). Gynoid fat mass was modestly lower in the high-adherence group (median 11.710 vs. 13.285 kg; *p* = 0.026). Regarding skeletal parameters, lumbar spine BMD was higher in the high-adherence group (median 0.98 vs. 0.88 g/cm^2^; *p* = 0.012), whereas total body BMD showed a borderline difference (*p* = 0.066), and femur BMD did not differ (*p* = 0.95) (Table 2).

As shown in Table 3, metabolic parameters were largely similar between MD adherence groups: total cholesterol, LDL, HDL, triglycerides, fasting glucose, insulin, and the HOMA index did not differ significantly (all *p* > 0.05). CAP values and steatosis grading were comparable between adherence strata (CAP *p* = 0.55; steatosis grading *p* = 0.53), and liver stiffness showed no significant difference (median 4.3 vs. 5.2 kPa; *p* = 0.12). Fibrosis stages and non-invasive fibrosis scores were also similar with respect to adherence (fibrosis stage *p* = 0.67; FIB-4 *p* = 0.35; APRI *p* = 0.09).

When stratified by geographic region, participants from Northern Italy had higher total cholesterol (median 187.5 vs. 168.0 mg/dL; *p* = 0.01), higher fasting glucose (median 103 vs. 95.0 mg/dL; *p* < 0.01), and a lower apoA/apoB ratio (median 0.61 vs. 0.68; *p* = 0.02) compared with those from Southern Italy (Table 3b). Liver stiffness was slightly higher in Southern Italy (median 5.6 vs. 4.6 kPa; *p* = 0.03), whereas CAP, steatosis grading, fibrosis staging, FIB-4 and APRI did not differ significantly by region (all *p* > 0.05).

In multivariable linear regression models adjusted for age, sex, and CD4^+^ nadir (Table 4), high MD adherence remained independently associated with a lower BMI (β −5.3; 95% CI −7.3 to −3.4; *p* < 0.001). In contrast, associations between MD adherence and BMD or body fat percentage were not statistically significant after adjustment. Geographic origin (north vs. south) was not independently associated with BMI, BMD, or body fat percentage in adjusted analyses.

Because ethnicity was nearly completely collinear with MD adherence group, it could not be entered as an independent covariate in these models. A sensitivity analysis restricted to Caucasian participants only confirmed associations consistent in direction and magnitude with the full cohort for all three outcomes (Supplementary Table S3).

We tested whether current smoking confounds the association between MD adherence and BMI by adding current smoking status to the multivariable model. The coefficient for high MD adherence on BMI changed minimally (β = −5.32, *p* < 0.001 without smoking vs. β = −5.17, *p* < 0.001 with smoking added; 2.75% relative change) and current smoking was not independently associated with BMI in the adjusted model (*p* = 0.45), Supplementary Table S4.

Similarly, we tested whether it confounded the association between MD adherence and BMI by adding physical activity status to the multivariable model. The coefficient for high MD adherence on BMI was unchanged (β = −5.31, *p* < 0.001 vs. β = −5.32, *p* < 0.001 without physical activity; 0.19% relative change) and physical activity was not independently associated with BMI in the adjusted model (*p* = 0.28), Table S5.

## 4. Discussion

In this cross-sectional study of ageing PWH with a stable virological suppression followed at two Italian centres, high adherence to the MD was common and was associated with a distinctly more favourable anthropometric and DXA-derived body composition profile. Specifically, participants with high MD adherence had markedly lower body weight and BMI and exhibited lower total fat mass, with the most consistent differences observed in central adiposity (lower trunk fat and android fat, in both grams and percentage). These findings are clinically relevant because central fat accumulation (including visceral and android depots) is strongly linked to insulin resistance, cardiometabolic risk, and adverse outcomes, and BMI alone may underestimate this risk in people with HIV [22,23]. Our results align with prior evidence in HIV suggesting that MD adherence is associated with a more favourable cardiometabolic profile and may mitigate ART-related metabolic complications [16,24]. In a cohort of people with ART-associated metabolic syndrome/lipodystrophy, higher MD adherence was associated with more favourable metabolic parameters, supporting a role for diet quality in metabolic health among people with HIV [16]. Observational work has also linked Mediterranean-style patterns to fewer body-shape abnormalities in people with HIV on ART, consistent with the direction of our DXA findings on central adiposity [25]. Beyond observational data, mechanistic and interventional evidence suggests that Mediterranean dietary patterns may improve immune–metabolic pathways relevant to HIV, including metabolic parameters, immune activation and gut microbiota composition [26]. Collectively, these data support biological plausibility: MD patterns—rich in fibre, unsaturated fats and polyphenols—may influence insulin sensitivity, lipid handling, inflammatory tone and gut–immune interactions, all of which are relevant in people with HIV who, despite an effective ART, may still experience residual inflammation and metabolic dysregulation [26].

Notably, waist circumference did not differ significantly between adherence groups despite the marked differences observed in DXA-derived trunk and android fat. We considered whether differences in body frame or lean mass could have accounted for this apparent discrepancy. Although participants with low Mediterranean diet adherence had slightly greater lean mass, consistently with their higher overall body weight, this difference was modest relative to the substantially larger differences in regional fat mass quantified by DXA. Likewise, height differed only marginally between groups. These findings suggest that body frame and lean tissue are unlikely to fully explain the lack of a significant difference in waist circumference. In this context further analyses considering appendicular lean mass index or lean mass adjusted for height/body size would have provided a more meaningful interpretation. Waist circumference represents an indirect anthropometric surrogate of abdominal adiposity and is influenced by several factors beyond fat accumulation, including abdominal musculature, skeletal frame, respiratory phase, visceral contents, bloating, posture, and measurement variability. In contrast, DXA-derived regional adiposity, including android and trunk fat allowed a more sensitive characterisation of central adiposity, as well as a more granular ability to better detect clinically meaningful differences in different compartments [27]. Our findings therefore reinforce the concept that DXA-derived measurements may identify clinically meaningful differences in fat distribution that are not adequately captured by conventional anthropometric indices alone, particularly in people living with HIV, in whom alterations in body composition may occur independently of changes in waist circumference. From a clinical standpoint, this also reinforces the value of integrating objective body composition assessments (where available) into risk stratification in ageing people with HIV, particularly when evaluating lifestyle interventions.

We also observed higher lumbar spine BMD among participants with high MD adherence in unadjusted comparisons; however, this association was attenuated in adjusted models, suggesting confounding by demographic or HIV-related variables. This pattern is plausible given that bone health in people with HIV is very multifactorial—driven by age, sex, smoking, vitamin D status, physical activity, ART history and comorbidities—and dietary patterns may exert modest effects that are difficult to disentangle in cross-sectional analyses [28,29]. Nonetheless, the direction of effect is coherent with the broader literature linking healthier dietary patterns to skeletal outcomes and merits further evaluation in longitudinal studies with more detailed bone-related covariates and fracture endpoints [30].

A further important finding is that MD adherence clustered with social and clinical determinants, which are also well-known determinants of important metabolic conditions [31]. High adherence was strongly associated with Caucasian ethnicity, higher education, and higher current CD4 metrics and differed by the region of enrolment. Figure 1 suggests that the variability in food group scores was driven mainly by region (meat, unrefined cereals, olive oil, vegetables) and, even more prominently, by ethnicity across multiple components. These observations underscore that dietary patterns may have been shaped by cultural, socioeconomic and contextual factors that may also influence access to care, health literacy, and comorbidity burden [32]. Therefore, dietary counselling in HIV care should be culturally tailored and embedded within broader strategies addressing social determinants of health. Furthermore, it would be interesting for future research to correlate this information with the quality of life of people living with HIV, which was found to be low in a previous study in Southern Italy [33].

Interestingly, current smoking was more prevalent among those with high MD adherence, highlighting that healthy diet and other risk behaviours may not cluster uniformly. This finding reinforces the need for integrated lifestyle interventions rather than single-domain counselling, even if smoking cessation has been recently shown to be poorly recommended and very difficult to implement across Italy [34]. Contemporary HIV guidelines emphasise comprehensive risk reduction, including smoking cessation, diet optimisation and physical activity, as pillars of cardiometabolic prevention in PWH [35]. Sensitivity analyses confirmed that smoking and physical activity did not confound the MD adherence and BMI association (Supplementary Tables S4 and S5).

Despite substantial differences in adiposity, we did not detect significant differences in routine lipid and glucose homeostasis markers by MD adherence. Several explanations are possible: (i) limited statistical power, especially given the smaller low-adherence subgroup; (ii) the pharmacologic management of dyslipidaemia or diabetes attenuating between-group differences; (iii) the possibility that diet quality influences adiposity earlier than it translates into detectable differences in fasting markers; and/or (iv) heterogeneity in ART regimens, duration and prior exposure. Similarly, CAP-derived steatosis and liver stiffness did not differ significantly by adherence, and only small regional differences in stiffness were observed. Given the smaller low-adherence subgroup, we performed a post hoc power analysis for hepatic outcomes: this study had 80% power to detect only medium-to-large effect sizes (Cohen’s d = 0.63 for continuous parameters CAP, liver stiffness, FIB-4, and APRI; Cohen’s w = 0.27–0.29 for categorical outcomes such as steatosis grading and fibrosis stage), well above the differences actually observed between groups (Table 3). These null findings should therefore be regarded as inconclusive rather than as evidence of no association. This is notable because MASLD/NAFLD is common in people with HIV and is linked to visceral adiposity and metabolic risk [36,37]. In the general population, Mediterranean dietary interventions can improve hepatic steatosis and NAFLD-related outcomes, suggesting that diet could be relevant for liver health; however, our cross-sectional data do not support a clear association between MD adherence and elastography-derived liver outcomes in this cohort [38]. Longitudinal designs with quantitative liver fat measures and careful adjustment for adiposity, alcohol intake and metabolic therapies will be needed to clarify whether MD adherence independently influences hepatic outcomes in people with HIV.

In our study, adherence to MD, as expected considering the historical origins of this dietary pattern, was higher in the South. However, contemporary evidence suggests that the traditional north–south gradient in dietary habits has become less pronounced over recent decades. National surveillance data from the Italian National Institute of Statistics and the PASSI (Progressi delle Aziende Sanitarie per la Salute in Italia, Nutrizione e consumo di frutta e verdura, Epicentro, Istituto Superiore di Sanità) surveillance system indicate that indicators of healthy eating, including regular fruit and vegetable consumption, are often comparable or even more favourable in northern than southern regions of Italy [39]. These trends have been attributed to evolving socioeconomic, educational, and lifestyle factors that have progressively modified traditional dietary patterns across the country. Therefore, the regional differences observed in our cohort likely reflect these broader changes rather than a contradiction of the Mediterranean diet’s cultural origins. Nevertheless, because recruitment site may also capture unmeasured demographic or socioeconomic characteristics, residual confounding cannot be excluded and should be considered when interpreting these findings.

This study has several limitations. First, its cross-sectional nature precludes causal inference and is vulnerable to reverse causality (e.g., individuals with obesity may report dietary changes). Second, the low-adherence group was relatively small, limiting power for some outcomes and adjusted analyses. Indeed, although the overall sample size provided sufficient precision for the primary BMI analysis, the substantial imbalance between the high-adherence and low-adherence groups resulted in limited statistical power for multivariable analyses of secondary outcomes; therefore, non-significant findings for measures such as BMD, body fat percentage, and metabolic markers should be interpreted cautiously, as clinically meaningful associations may have gone undetected. Third, residual confounding is likely, particularly because MD adherence was highly associated with ethnicity and education; additional unmeasured factors (income, food insecurity, detailed physical activity intensity, total caloric intake, sleep, depression, current recreational drug use, including cocaine, opioids, and cannabis, which can potentially affect body weight and composition) could contribute to observed associations. In particular, a sensitivity analysis restricted to Caucasian participants only yielded associations between MD adherence and BMI, BMD, and body fat percentage that were consistent in direction and magnitude with those in the full cohort, arguing against ethnicity as the principal driver of these associations; nonetheless, residual confounding by ethnicity or related unmeasured sociocultural factors cannot be entirely excluded. Also, dietary assessment relied on questionnaire-based scoring and may be affected by reporting bias. Lastly, the study setting was limited to two specialised HIV clinics in Italy, and the inclusion of people with stable virological suppression may limit the generalisability of the results.

Within these constraints, our findings support the hypothesis that higher adherence to an MD pattern is associated with lower central adiposity and lower BMI in ageing people with HIV with stable virological suppression. Future prospective and interventional studies are warranted to determine whether improving adherence to a Mediterranean dietary pattern may favourably influence body composition, metabolic parameters, and the burden of HIV-associated comorbidities, ultimately helping to define the role of dietary interventions as part of comprehensive long-term HIV care.

## Figures and Tables

**Table 1:** Clinical characteristics of study population by Mediterranean diet adherence.

Characteristic	Overall (*n* = 130)	Low Adherence (*n* = 25)	High Adherence (*n* = 105)	*p*-Value *
Demographics and lifestyle				
Place (north), *n* (%)	78 (60)	22 (88)	56 (53.3)	0.001
Place (south), *n* (%)	52 (40)	3 (12)	49 (46.7)	0.001
Ethnicity other than Caucasian, *n* (%)	10 (7.7)	10 (40)	0 (0)	<0.001
Age, years, median (IQR)	57.4 (48.3–69.25)	60.6 (48.2–70.5)	57.4 (48.7–69.1)	0.95
Male sex, *n* (%)	106 (81.5)	20 (80)	86 (81.9)	0.78
High education level, *n* (%)	46 (35.4)	4 (16)	42 (40)	0.024
Current smokers, *n* (%)	62 (47.7)	7 (28)	55 (52.4)	0.047
Regular physical exercise, *n* (%)	43 (33.1)	8 (32)	35 (33.3)	0.90
Alcohol use disorder, *n* (%)	39 (30)	10 (40)	29 (27.6)	0.22
HIV-related variables				
Risk factor for HIV acquisition				0.83
Condomless sex between men	89 (68.5)	19 (76)	70 (66.7)	
Condomless heterosexual sex	25 (19.2)	3 (12)	22 (21)	
Intravenous drug use	9 (6.9)	2 (8)	7 (6.7)	
Other	7 (5.3)	1 (4)	6 (5.7)	
Years living with HIV, median (IQR)	15 (10–20)	12 (8–17)	15 (10–21)	0.10
Prior AIDS event, *n* (%)	20 (15.4)	3 (12)	17 (16.2)	0.76
CD4^+^ T cell at nadir, cells/µL, median (IQR)	270 (120–430)	199 (89–315)	298 (126.5–431.5)	0.17
Current CD4^+^ T cell, cells/µL, median (IQR)	784.5 (617–1039)	636 (558–871)	832 (642–1079)	0.021
CD4/CD8 ratio, median (IQR)	0.96 (0.7–1.3)	0.75 (0.6–0.9)	1.05 (0.8–1.4)	0.018
Antiretroviral regimen				0.77
2NRTI+INI	64 (49.2)	9 (36)	55 (52.4)	
2NRTI+NNRTI	33 (25.4)	8 (32)	25 (23.8)	
NRTI+INI	25 (19.2)	7 (28)	18 (17.1)	
2NRTI+PI	4 (3.1)	1 (4)	3 (2.9)	
Other	4 (3.1)	0 (0)	4 (3.8)	
Comorbidities and polypharmacy				
Chronic kidney disease, *n* (%)	6 (4.6)	1 (4)	5 (4.8)	0.43
Chronic obstructive pulmonary disease, *n* (%)	37 (28.5)	6 (24)	31 (29.5)	0.58
Diabetes, *n* (%)	12 (9.2)	5 (20)	7 (6.7)	0.054
Dyslipidaemia, *n* (%)	48 (36.9)	9 (36)	39 (37.1)	0.92
Ischemic heart disease, *n* (%)	16 (12.3)	3 (12)	13 (12.4)	>0.99
Hypertension, *n* (%)	32 (24.6)	6 (24)	26 (24.8)	0.94
Obesity, *n* (%)	37 (28.5)	18 (72)	19 (18.1)	<0.001
Neurological disorders, *n* (%)	4 (3.1)	3 (12)	1 (1)	0.022
Multimorbidity, *n* (%)	57 (43.8)	10 (40)	47 (44.8)	0.67
Polypharmacy, *n* (%)	20 (15.4)	5 (20)	15 (14.3)	0.54
Use of statins, *n* (%)	30 (23.1)	5 (20.0)	25 (23.8)	0.68
Use of anti-hypertensive agents, *n* (%)	25 (19.2)	3 (12.0)	22 (21.0)	0.40
Use of anti-diabetic agents, *n* (%)	9 (6.9)	4 (16.0)	5 (4.8)	0.07

* Comparisons used χ^2^/Fisher’s exact test or Wilcoxon rank-sum test, as appropriate.

**Table 2:** Anthropometrics and DXA parameters by Mediterranean diet adherence.

Parameter	Overall (N = 130)	Low Adherence (*n* = 25)	High Adherence (*n* = 105)	*p*-Value *
Weight, kg, median (IQR)	77.0 (68.0–87.0)	88.0 (80.0–93.0)	74.4 (65.5–82.5)	<0.001
Height, cm, median (IQR)	170.0 (164.0–174.0)	165.0 (162.0–171.0)	170.0 (165.0–175.0)	0.057
BMI, kg/m^2^, median (IQR)	26.62 (23.29–30.40)	31.38 (29.75–33.91)	25.59 (22.68–28.73)	<0.001
Waist circumference, cm, median (IQR)	97.0 (90.0–108.0)	98.0 (91.0–110.0)	96.5 (90.0–108.0)	0.51
Total BMD, g/cm^2^, median (IQR)	1.10 (1.05–1.23)	1.05 (1.01–1.17)	1.10 (1.05–1.23)	0.066
Femur BMD, g/cm^2^, median (IQR)	0.80 (0.70–0.93)	0.79 (0.70–0.97)	0.80 (0.72–0.89)	0.95
Lumbar spine BMD, g/cm^2^, median (IQR)	0.93 (0.87–1.07)	0.88 (0.84–0.89)	0.98 (0.88–1.08)	0.012
Total fat mass, kg, median (IQR)	18.962 (16,500–24,832)	21.921 (18.962–33.872)	18.606 (16.500–24.097)	0.009
Trunk fat mass, kg, median (IQR)	11.546 (9.686–15.934)	15.934 (11.546–17.897)	11.471 (9.602–15.721)	0.002
Trunk fat, %	31.60 (27.50–35.00)	35.00 (32.80–42.60)	31.10 (25.60–33.60)	0.004
Android fat, kg, median (IQR)	1.930 (1.762–3.101)	3.107 (1.930–3.490)	1.930 (1.630–2.650)	0.001
Android fat, %	33.20 (30.70–39.80)	39.80 (35.60–45.70)	33.20 (29.70–36.90)	0.001
Gynoid fat, kg, median (IQR)	12.375 (10.244–13.823)	13.285 (12.375–13.823)	11.710 (10.244–13.823)	0.026
Total lean mass, kg, median (IQR)	52.1 (48.0–56.8)	55.8 (51.7–61.3)	51.2 (47.5–55.6)	0.09

* Comparisons used Wilcoxon rank-sum test.

**Table 3:** Metabolic and liver parameter assessment by adherence (**a**) and place (**b**).

**(a) Metabolic and liver parameter assessment. Comparison between low adherence and high adherence.**			
**Parameter**	**Low Adherence (*n* = 25)**	**High Adherence (*n* = 105)**	***p*** -Value *
BMI, kg/m^2^ median (IQR)	31.38 (29.75–33.91)	25.59 (22.68–28.73)	<0.01
Total cholesterol, mg/dL, median (IQR)	178 (149–199)	176 (153–197)	0.87
LDL, mg/dL, median (IQR)	119 (81–136)	111 (82–125)	0.60
HDL, mg/dL, median (IQR)	42 (35–46)	42 (37–51)	0.18
Triglycerides, mg/dL, median (IQR)	123 (102–151)	123 (90–212)	0.97
apoA/apoB ratio, median (IQR)	0.61 (0.50–0.69)	0.65 (0.49–0.80)	0.78
Fasting glucose, mg/dL, median (IQR)	103 (89–106)	98 (89–108)	0.54
Insulin, µIU/mL, median (IQR)	12 (10–18)	11 (8–19)	0.51
HOMA index, median (IQR)	1.9 (1.2–2.7)	2.2 (1.3–4.2)	0.27
CAP, median (IQR)	248 (232–281)	244 (187–289)	0.55
Steatosis grading, n (%)			0.53
None	11 (44)	54 (51.4)	
S1	4 (16)	10 (9.5)	
S2	3 (12)	7 (6.7)	
S3	7 (28)	34 (32.4)	
Liver stiffness (kPa), median, IQR	4.3 (3.5–6.3)	5.2 (4.0–6.5)	0.12
Liver fibrosis, n (%)			0.67
F0-F1	23 (92)	90 (85.7)	
F2	1 (4)	11 (10.5)	
F3	1 (4)	4 (3.8)	
F4	0 (0)	0 (0)	
FIB-4 index, median, IQR	0.65 (0.46–0.86)	0.66 (0.45–1.35)	0.35
APRI score, median IQR	0.23 (0.16–0.29)	0.25 (0.20–0.36)	0.09
**(b) Metabolic and liver parameter assessment. Comparison between northern people and southern people.**			
**Parameter**	**People from the North (*n* = 78)**	**People from the South (*n* = 52)**	***p*** -Value *
BMI, Kg/m^2^ median (IQR)	27.49 (23.81–31.22)	25.39 (22.69–29.21)	0.01
Total cholesterol, mg/dL, median (IQR)	187.5 (155.0–205.0)	168.0 (143.0–185.5)	0.01
LDL, mg/dL, median (IQR)	108.5 (74.0–124.0)	116.0 (95.0–137.5)	0.08
HDL, mg/dL, median (IQR)	42.0 (36.0–51.0)	43.0 (37.5–50.5)	0.87
Triglycerides, mg/dL, median (IQR)	123 (92–203)	134 (85–213)	0.94
apoA/apoB ratio, median (IQR)	0.61 (0.49–0.73)	0.68 (0.51–0.88)	0.02
Fasting glucose, mg/dL, median (IQR)	103 (91–111)	95.0 (88.0–101.5)	<0.01
Insulin, µIU/mL, median (IQR)	12.0 (9.0–19.0)	10.5 (6.5–18.0)	0.06
HOMA index, median (IQR)	2.1 (1.2–3.0)	2.4 (1.6–4.3)	0.19
CAP, median (IQR)	240 (202–294)	255 (216–288)	0.47
Steatosis grading			0.71
None	41 (52.6%)	24 (46.2%)	
S1	8 (10.3%)	6 (11.5%)	
S2	7 (9.0%)	3 (5.8%)	
S3	22 (28.2%)	19 (36.5%)	
Liver stiffness (KPa), median, IQR	4.6 (3.7–6.3)	5.6 (4.2–7.3)	0.03
Liver fibrosis			0.66
F0-F1	69 (88.5%)	44 (84.6%)	
F2	7 (9.0%)	5 (9.6%)	
F3	2 (2.6%)	3 (5.8%)	
F4	0 (0%)	0 (0%)	
FIB-4 index, median, IQR	0.73 (0.48–1.28)	0.60 (0.40–1.15)	0.61
APRI score, median IQR	0.28 (0.19–0.36)	0.22 (0.18–0.31)	0.25

* Comparisons used χ^2^/Fisher’s exact test or Wilcoxon rank-sum test, as appropriate.

**Table 4:** Multivariable linear regression models adjusted for age, sex, and CD4^+^ nadir.

	High MD Adherence			Geographic Origin (North vs. South)		
	Adjusted β	95% CI	*p*-Value	Adjusted β	95% CI	*p*-Value
**Body fat percentage**	−0.03	−0.06, 0.00	0.054	0.00	−0.03, 0.03	0.99
**BMD**	0.04	−0.01, 0.09	0.10	−0.01	−0.07, 0.04	0.60
**BMI**	−5.32	−7.30, −3.40	<0.001	−2.10	−4.30, 0.01	0.051

Abbreviation: CI = Confidence Interval; BMD = Bone Mineral Density; BMI = Body Mass Index.

## Data Availability

All data used to produce this manuscript are available upon reasonable request to the corresponding author.

## Supplementary Materials

The following supporting information can be downloaded at: https://www.mdpi.com/article/10.3390/v18090931/s1, Table S1: Questionnaire assessing weekly food consumption frequency (MedDiet Score); Table S2: Alcohol consumption by MD adherence, region and ethnicity; Table S3. Multivariable linear regression models of MD adherence adjusted for age, sex, and CD4^+^ nadir in cohort of Caucasian only (N = 120); Table S4: Sensitivity analysis: current smoking as a potential confounder of the MD adherence–BMI association; Table S5: Sensitivity analysis: physical activity as a potential confounder of the MD adherence–BMI association.
