# Supplementary material for: Eating Habits and Possible Effects of Mediterranean Diet on Body Composition in Ageing People Living with HIV in Two Italian Centres
Source: Viruses. 2026 Aug 25;18(9):931. doi: 10.3390/v18090931 (PMC13611921; doi:10.3390/v18090931)
Supplement: Supplementary file 1 [file viruses-18-00931-s001.zip › viruses-4441713-supplementary.pdf]

Table S1. Questionnaire assessing weekly food consumption frequency (MedDiet Score).

Food	Weekly Consumption Frequency (Times/Week)					
Unrefined cereals (pasta, rice, bread, etc.)	Never	1–6	7–12	13–18	19–31	≥32
Score	0	1	2	3	4	5
Potatoes	Never	1–4	5–8	9–12	13–18	>18
Score	0	1	2	3	4	5
Fruits	Never	1–4	5–8	9–15	16–21	≥22
Score	0	1	2	3	4	5
Vegetables	Never	1–6	7–12	13–20	21–32	>32
Score	0	1	2	3	4	5
Legumes	Never	<1	1–2	3–4	5–6	>6
Score	0	1	2	3	4	5
Fish	Never	<1	1–2	3–4	5–6	>6
Score	0	1	2	3	4	5
Red meat and derivatives	≤1	2–3	4–5	6–7	8–10	>10
Score	5	4	3	2	1	0
Poultry	≤3	4–5	5–6	7–8	9–10	>10
Score	5	4	3	2	1	0
Dairy products (milk, yoghurt, cheese)	≤10	11–15	16–20	21–28	29–30	>30
Score	5	4	3	2	1	0
Olive oil	Never	Rarely	<1	1–3	3–5	Always
Score	0	1	2	3	4	5
Alcohol consumption, ml/die,	<300 mL (1–2 glasses)/none	300 mL (3 glasses)	400 mL (4 glasses)	500 mL (5 glasses)	600 mL (6 glasses)	≥700 mL (7 glasses or more) or never
Score	5	4	3	2	1	0

Table S2: Alcohol consumption by MD adherence, region and ethnicity.

Alcohol intake	Overall (N=130)	Low Adherence (N=25)	High Adherence (N=105)	p-value	Northern people (N=78)	Southern people (N=52)	p-value	Caucasian (N=120)	No Caucasian (N=10)	p-value
No consumption, n (%)	41 (32)	17 (68)	24 (23)	**<0.001**	27 (35)	14 (27)	0.50	36 (30)	5 (50)	**0.012**
Low consumption, n (%)	18 (14)	4 (16)	14 (13)		11 (14)	7 (13)		14 (12)	4 (40)	
Moderate consumption, n (%)	25 (19)	4 (16)	21 (20)		13 (17)	12 (23)		24 (20)	1 (10)	

High-risk consumption, n (%)	46 (35)	0 (0)	46 (44)		27 (35)	19 (37)		46 (38)	0 (0)	

Table S3. Multivariable linear regression models of MD adherence adjusted for age, sex, and CD4+ nadir in cohort of Caucasian only (N=120).

	High MD adherence		
	**Adjusted β**	**95% CI**	**p-value**
**Body fat percentage**	-0.04	-0.08, -0.01	0.034
**BMD**	0.06	-0.01, 0.12	0.088
**BMI**	-5.90	-8.30, -3.50	<0.001

Table S4: Sensitivity analysis: current smoking as a potential confounder of the MD adherence–BMI association.

	Beta	95% CI	p-value
High adherence	-5.2	-7.2, -3.2	<0.001
Age	-0.05	-0.11, 0.00	0.047
Sex	-2.4	-4.4, -0.41	0.019
CD4 nadir	0.0	0.0, 0.0	0.60
Smoking	-0.6	-2.2, 0.98	0.45

Table S5: Sensitivity analysis: physical activity as a potential confounder of the MD adherence–BMI association.

	Beta	95% CI	p-value
High adherence	-5.3	-7.2, -3.4	<0.001
Age	-0.06	-0.11, 0.00	0.035
Sex	-2.3	-4.3, -0.36	0.021
CD4 nadir	0.0	0.0, 0.0	0.60
Physical activity	-0.89	-2.5, 0.73	0.28
